# Biotechnological production of hyaluronic acid: a mini review

**DOI:** 10.1007/s13205-016-0379-9

**Published:** 2016-02-15

**Authors:** Jun Hui Sze, Jeremy C. Brownlie, Christopher A. Love

**Affiliations:** 1 School of Natural Sciences, Griffith University, Nathan, QLD 4111 Australia; 2 Environmental Futures Research Institute, Griffith University, Nathan, QLD 4111 Australia; 3 Eskitis Institute for Drug Discovery, Griffith University, Nathan, QLD 4111 Australia

**Keywords:** Hyaluronic acid, HA synthase, *Streptococcus*, Microbial production

## Abstract

Hyaluronic acid (HA) is a polysaccharide found in the extracellular matrix of vertebrate epithelial, neural and connective tissues. Due to the high moisture retention, biocompatibility and viscoelasticity properties of this polymer, HA has become an important component of major pharmaceutical, biomedical and cosmetic products with high commercial value worldwide. Currently, large scale production of HA involves extraction from animal tissues as well as the use of bacterial expression systems in *Streptococci*. However, due to concerns over safety, alternative sources of HA have been pursued which include the use of endotoxin-free microorganisms such as *Bacilli* and *Escherichia coli*. In this review, we explore current knowledge of biosynthetic enzymes that produce HA, how these systems have been used commercially to produce HA and how the challenges of producing HA cheaply and safely are being addressed.

## Introduction

Hyaluronic acid (HA) is a linear glycosaminoglycan polymer commonly found in the extracellular matrix of vertebrate epithelial, neural and connective tissues. It is also involved in many signalling pathways including those involved in embryonic development, wound healing, inflammation and cancer (Stern et al. 2006). HA is also a component of the extracellular capsule formed by some microorganisms, such as *Streptococcus*, that serves not only for adherence and protection, but also as a molecular mimicry to evade host’s immune system during its infection process (Wessels et al. 1991).

Due to its high moisture retention and viscoelasticity properties, numerous biomedical, pharmaceutical and cosmetic products have been developed with the use of HA. Depending on the type and function of the HA-based end-product, the starting material is dependent on the chain length of HA to define its application. For example, HA with high molecular weight (greater than 10 kDa) is desirable for products used in ophthalmology, orthopaedic, cosmetics and tissue engineering (Allison and Grande-Allen 2006; Fagien and Cassuto 2012; Kogan et al. 2007) whereas HA with low molecular weight (about 5 kDa and below) are useful for producing substances that promote angiogenesis, inhibit tumour progression or induce expression of pro-inflammatory mediators (Jagannath and Ramachandran 2010; Tammi et al. 2008).

Currently there are two production processes employed to obtain HA polymer in commercial quantities: extraction from animal tissues, typically rooster combs, or more recently though the application of bacterial expression systems in *Streptococcus*. Both approaches have faced considerable concerns over the safety of using biomedical products derived either from animal products or *Streptococcus*, a known pathogen that produces several endotoxins (Liu et al. 2011). To address these issues, two alternatives have been considered: (a) to generate HA via cell-free system, and thus avoid contamination of endotoxins and reduce cost of purification and/or (b) genetically engineer different microorganisms that do not produce endotoxins (Widner et al. 2005; Yu and Stephanopoulos 2008). In this review, we explore current knowledge of biosynthetic enzymes that produce HA, how these systems have been used commercially to produce HA and discuss how the challenges of producing HA safely and cheaply are being addressed.

## Hyaluronic acid

**Fig. 1:**
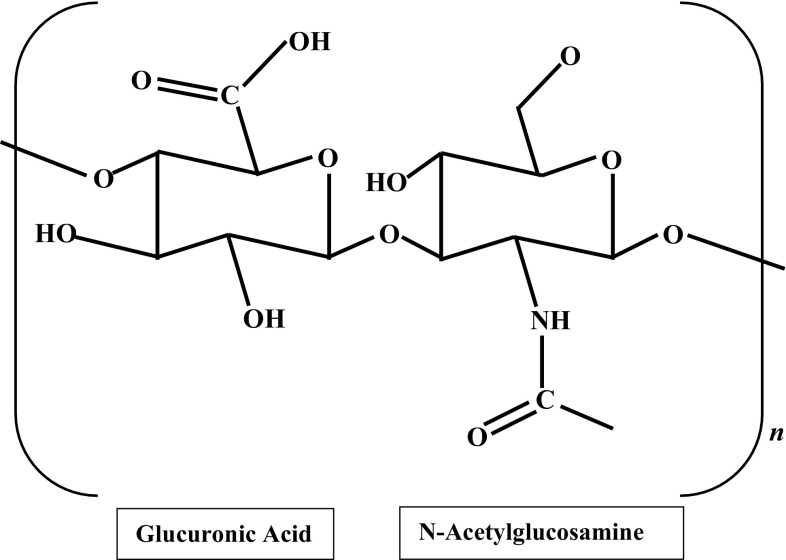
Structure of a hyaluronic acid monomer. HA consists of glucuronic acid and *N*-acetylglucosamine that can be repeated up to 10,000 times or more (Cowman and Matsuoka 2005)

Hyaluronic acid (HA), also known as hyaluronan, is a linear glycosaminoglycan (GAG) composed of repeating disaccharides of β4-glucuronic acid (GlcUA)-β3-*N*-acetylglucosamine (GlcNAc) (Fig. 1). HA was first isolated and identified from the vitreous body of bovine’s eye (Meyer and Palmer 1934). Eventually this polysaccharide was found to be ubiquitously distributed in many parts of vertebrate tissues (brain, umbilical cord, synovial fluid in between joints, skin, rooster comb, neural tissues and epithelium) with differing concentrations and molecular weights (Fraser et al. 1997).

HA is naturally negatively charged (due to the carboxylate groups) and this allows it to bind to a large amount of water forming a highly viscous gel. This anionic gel-like polymer offers not only lubrication for the joints but also acts as a shock-absorber for the surrounding tissues (Fraser et al. 1997). Moreover, it is non-immunogenic as it does not initiate immune response in humans and other vertebrates (Schiraldi et al. 2010). Due to its unique biocompatibility and viscoelastic properties, it lends itself to biomedical applications such as drug delivery, ophthalmic surgery, osteoarthritis treatment and tissue engineering (DeAngelis 2012). It is also used in cosmetics applications, notably as dermal fillers and moisturizers (Fagien and Cassuto 2012). HA is recognised as a signalling molecule involved in many mammalian biological processes and plays critical roles in several disease causing events such as inflammation, tumorigenesis and abnormal immune function (Cooper et al. 2008; Stern et al. 2006; Tammi et al. 2008). As HA does not initiate an immune response, bacteria such as *Streptococcus zooepidemicus* synthesise HA as a means to encapsulate their cells and exhibit molecular mimicry to escape detection from the host’s immune system (Boyce et al. 2000; Wessels et al. 1991). HA is also naturally produced by other pathogenic bacteria including *Streptococcus pyogenes, Streptococcus uberis, Pasteurella multocida* and *Cryptococcus neoformans* (Blank et al. 2008; DeAngelis et al. 1993, 1998; Jong et al. 2007).

## Biosynthesis of HA

### *has* operon in *Streptococci*

The *has* operon was first discovered in early 1990s when a gene encoding the enzyme responsible for HA synthesis, denoted as HA synthase or HAS, was identified from Group A *Streptococcus* (*S. pyogenes*) (DeAngelis et al. 1993). This gene is part of an operon containing the *hasA* gene that encodes HA synthase, *hasB* gene that encodes UDP-glucose dehydrogenase, and the *hasC* gene that encodes UDP-glucose pyrophosphorylase (Crater et al. 1995; Dougherty and van de Rijn 1993). The *has* operon in other streptococci, such as *S. zooepidemicus*, contain two additional genes, *hasD* and *hasE* that encode a bi-functional enzyme (acetyltransferase and pyrophosphorylase) and phosphoglucoisomerase respectively, allowing HA to be synthesised by additional metabolic pathway. The addition of these two genes to the *has* operon are thought to have been facilitated by intragenomic gene duplication together with frequent homologous recombination (Blank et al. 2008).

### Biosynthetic pathway

**Fig. 2:**
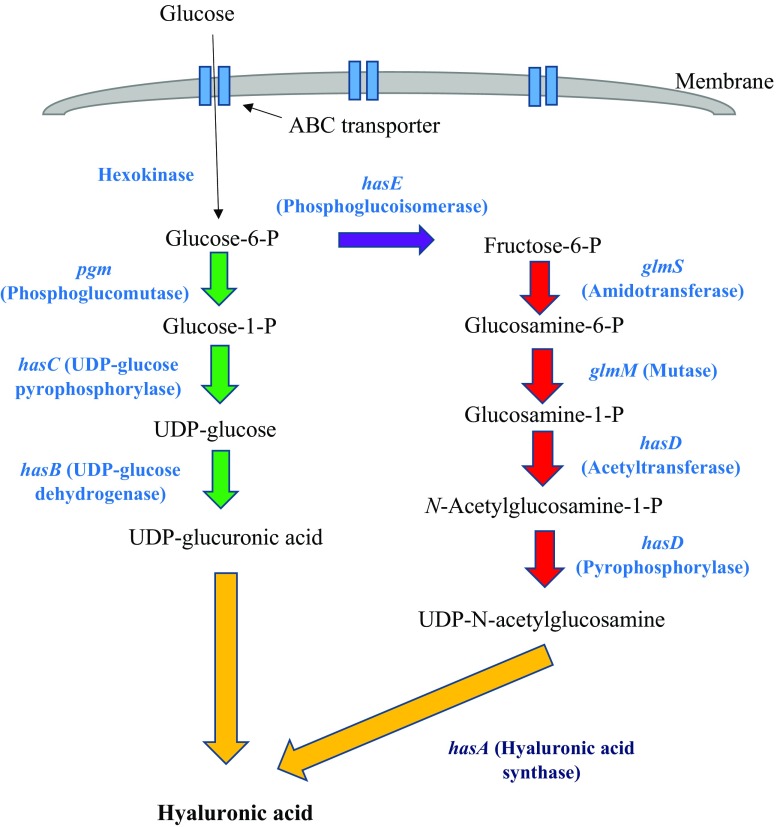
Hyaluronic acid biosynthetic pathway in *S. zooepidemicus*. Glucose is first converted by hexokinase to form glucose-6-Phosphate which then enters one of the two distinct pathways to form UDP-glucuronic acid (*pgm, hasC* and *hasB*) or UDP-*N*-acetylglucosamine (*hasE, glmS, glmM* and *hasD*). These precursors are then bound together via the action of hyaluronic acid synthase or HAS (encoded by *hasA* in *S. zooepidemicus*) to form hyaluronic acid

Most studies of HA biosynthesis in prokaryotes have focused on *S. zooepidemicus*, which uses two distinct pathways to synthesise HA precursors (Chong et al. 2005; DeAngelis 1996). Biosynthesis of HA begins with the phosphorylation of glucose by hexokinase to produce the main precursor, glucose-6-phosphate. From here, HA synthesis pathway can then be divided into two distinct pathways that synthesise the two building blocks of HA, glucuronic acid and *N*-acetylglucosamine (Fig. 2).

In the first set of reactions, α-phosphoglucomutase (*pgm*) converts glucose-6-phosphate to glucose-1-phosphate before a phosphate group from UTP is transferred to glucose-1-phosphate by UDP-glucose pyrophosphorylase (*hasC*) to produce UDP-glucose. Finally, UDP-glucose is oxidised by UDP-glucose dehydrogenase (*hasB*) to yield the first HA precursor, UDP-glucuronic acid (UDP-GlcUA). In the second set, glucose-6-phosphate is converted to fructose-6-phosphate by phosphoglucoisomerase (*hasE*). Once converted, fructose-6-phosphate is tagged with an amido group transferred from a glutamine residue via amidotransferase (*glmS*) to produce glucosamine-6-phosphate and then is modified by mutase (*glmM*) to yield glucosamine-1-phosphate. This intermediate is then sequentially acetylated and phosphorylated by acetyltransferase and pyrophosphorylase, respectively (*hasD*) to yield the second HA precursor, UDP-*N*-acetylglucosamine (UDP-GlcNAc). Once the two precursors are synthesised, hyaluronic acid/hyaluronan synthase (*hasA*) polymerises the two components in an alternate manner to produce the HA polymer. It is important to note that HA biosynthesis is an energy-consuming process for the bacteria as several intermediates are also used in cell wall biosynthesis, biomass formation and lactate formation via glycolysis (Shah et al. 2013).

## HA synthase (HAS)

In general, for any bacterium to synthesise HA capsule, the HAS gene must be present as it is needed to polymerize UDP-sugar precursors into HA. The *hasA* was first isolated from *S. pyogenes* from Group A *Streptococcus* (GAS) and it was shown to have the capability to direct HA capsule biosynthesis in acapsular mutants as well as heterologous bacteria such as *E. coli* and *Enterococcus faecalis* (DeAngelis et al. 1993). Since then, other HA synthase (HAS) related genes have been identified in other organisms including Group C *Streptococcus*, algae, viruses and vertebrates. Analysis of the primary sequence and predicted structural topologies have shown HAS enzymes share many common features and have been divided into two categories, (based on similarities and differences in amino acid motifs, topology and mode of action) designated as Class I and Class II HAS (Table 1).

**Table 1:** Characteristics and features of Class I and Class II HA synthases

Feature	Class I	Class II
Source organisms	Streptococci, amphibian, mammal, yeast, virus	*Pasteurella multocida*
Ability to be expressed as soluble active protein	No (must be associated with cell membrane)	Yes (amino acid residues 1–703)
Number of GT2 module	1	2
HA chain growth	At reducing end—Streptococcus, humans and mice At non-reducing end—*Xenopus laevis* and algal virus	At non-reducing end
Topology of the protein	Multiple membrane associated domains throughout the whole protein	Two catalytically independent domains, A1 and A2 attached to membrane via C-terminal region
Intrinsic polymerization	Processive	Non-processive
Size (amino acids)	417–588	972
Primer for initiation of HA synthesis?	No	Yes
Involvement of other molecules/proteins during HA translocation	Yes—involves lipid molecules (membrane bilayer)	Yes—may involve capsular polysaccharide transport machinery (more studies needed to confirm)

### Class I HAS

The vast majority of HAS enzymes are Class I and have been identified in Chlorella-like green algae virus (PBCV-1), Group A and C *Streptococci* as well as vertebrates (DeAngelis et al. 1993, 1997; Fraser et al. 1997). The key features that define Class I HAS enzymes include:

1. Class I HAS enzymes have a single glycosyltransferase 2 (GT2) family module (a subset of common catalytic residues among glycosyltransferase enzymes, for their sugar addition reactions) and share amino acid similarities in the central region of the protein sequence;
2. This protein group is an integral membrane enzyme with four to six predicted transmembrane domains and one to two membrane domains are found in its structure (Weigel 2002; Weigel and DeAngelis 2007);
3. These enzymes are thought to be associated with lipid molecules (Fig. 3a) in order to synthesise and extrude the HA molecules out of the cells to form either the capsule or to be released into the extracellular space (Tlapak-Simmons et al. 1999; Weigel 2015; Weigel and DeAngelis 2007) and

**Fig. 3:**
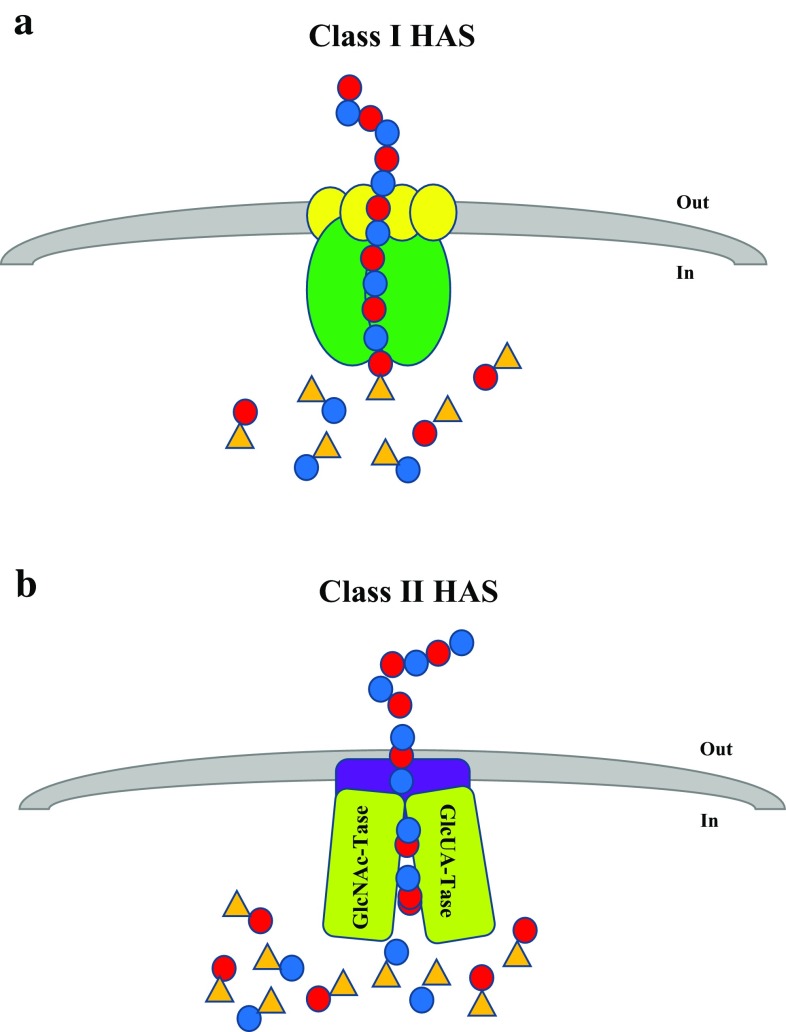
Schematic representation of HA biosynthesis in Class I and Class II Hyaluronic Acid Synthases (HAS). **a** Class I HAS are integral membrane proteins that catalyse the UDP-sugar addition to growing HA chain and may transport the hydrophilic HA polymer across the cell membrane of eukaryotes or Gram-positive bacteria. Lipid molecules (*yellow circles*) facilitate the HAS activity which allows it to direct HA translocation. **b** Class II HAS is a peripheral protein that also catalyses HA elongation. This enzyme is a hybrid of two glycosyltransferases that transfers GlcNAc-UDP and GlcUA-UDP at the non-reducing end of the HA chain. It was suggested that this protein may interact with other cell membrane proteins (capsular polysaccharide transport protein–*purple block*) in order to translocate HA across the cell membrane of Gram-negative bacteria (*P. multocida*). *Blue* and *red dots* represent GlcUA and GlcNAc, respectively. *Orange triangle* represents the UDP component of the sugar
4. Class I HAS adds UDP-sugars to the growing HA polymer from the reducing end (Fig. 4) of the growing HA chain (and some at the non-reducing end for *Xenopus laevis* and algal virus HAS) (Weigel 2002).

**Fig. 4:**
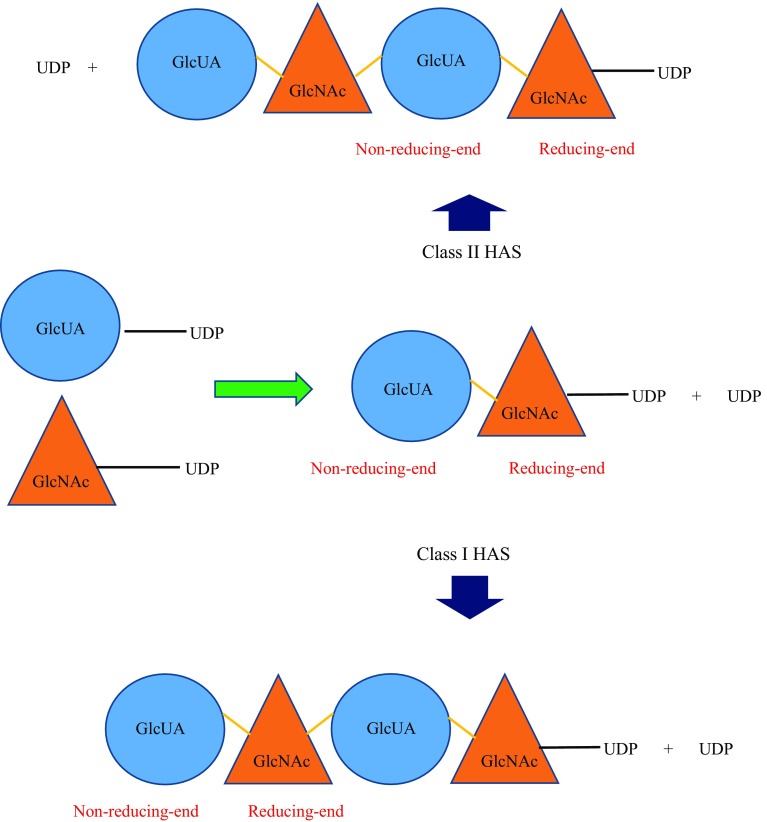
Position of non-reducing and reducing ends of a growing HA chain. Polymerisation of HA by Class I HAS occurs whereby the UDP group present at the reducing end of the HA polysaccharide is released to form a glycosidic bond between the growing chain and the new sugar-UDP (in this example GlcUA-UDP would be added, followed by GlcNAc-UDP). Class II HAS adds new sugar-UDPs to the non-reducing end of the HA polysaccharide. The HA growing chain remains attached to the same UDP group at the reducing end (throughout the whole polymerisation process) while subsequent new sugar-UDPs are being added to the non-reducing end. *Yellow line* represents the glycosidic bond formed between GlcUA and GlcNAc

### Class II HAS

The only organism known to encode for a Class II HAS enzyme, thus far, is *P. multocida* (DeAngelis et al. 1998). The protein (known as pmHAS) has a number of key features that distinguishes it from Class I HAS:

1. pmHAS is approximately twice the size of a Class I HAS (972 versus 417–588 residues, respectively) (DeAngelis et al. 1998);
2. pmHAS has two GT2 family modules (Weigel and DeAngelis 2007);
3. pmHAS is a peripheral membrane protein that does not necessarily require association with lipid bilayer and transporters of cell membrane to synthesise HA;
4. pmHAS consists of two independent catalytic domains that are responsible for GlcNAc and GlcUA transferase activity (Fig. 3b) (Jing and DeAngelis 2000); and
5. pmHAS adds UDP-sugars to the growing HA polymer from the non-reducing end (DeAngelis 1999) (Fig. 4)

pmHAS has been expressed and successfully synthesised HA in a number of bacterial species, including *E. coli* (Liu et al. 2011; Mao and Chen 2007; Mao et al. 2009). Since then this enzyme has become a focal point for cell-free HA production as its truncated form (pmHAS1-703) does not need to be bound to a membrane in order to synthesise HA (DeAngelis et al. 1998; Jing and DeAngelis 2003, 2004; Kooy et al. 2013).

## HA market in healthcare industry

HA’s essential functions in the human eye, synovial fluid of joints and in the epidermal layers, has led to considerable interest in developing new methods to successfully synthesise and deliver HA. A recent market analysis report predicted that as a consequence of an aging population and an increase in osteoarthritis the global market for HA visco-supplementation in humans alone was estimated to be more than $2.5 billion by 2017 (MRG.Net 2013).

The first ever patented HA product, called *Healon*, was obtained from rooster combs (Balazs 1979). This product was an immediate success following its use as a viscoelastic substance that acts to supplement and recover the loss of vitreous body fluid during eye surgery. The introduction of this non-inflammatory HA product has since led to the development of other HA-based products which are useful for biomedical and cosmetic applications.

The first ever single-injection HA viscosupplementation product, *Synvisc*-*One* and produced by Genzyme, was approved by the US Food and Drug Administration (FDA) in 2009 and has since gained a lot of popularity due to its convenience and effectiveness in relieving pain for osteoarthritis in knee joints. Following this major breakthrough, the demand for easy-to-use HA-based products has escalated in other parts of the world including Europe and the Asia-Pacific. *Restylane*, is the first HA-based dermal filler developed to correct moderate to severe wrinkles and folds. This product is not only effective but it is also widely used in more than 65 countries because of utilisation of production technology called NASHA (non-animal stabilized HA) which eliminates the use of animal parts during HA extraction (van Eijk and Braun 2007). Other HA-based products that are being sold in the current market are skin moisturizers, wound dressing materials (to promote wound healing after surgery) (Kogan et al. 2007) and polymeric scaffolds for controlled drug release and tissue engineering (Allison and Grande-Allen 2006).

## Current production of HA and potential concerns

Currently, industrial production of HA is based on either HA extraction from animal tissues or via large-scale bacterial fermentation with genetically modified strains. These processes are widely used and have been able to generate HA products with molecular weights above 1 MDa (as half-life of the molecule will increase and persist longer while maintaining its physiological function), which is desirable for biomedical and cosmetic use (Liu et al. 2011).

### Extraction from animal tissue

Since the early 1930s when HA was first isolated from bovine vitreous humor (Meyer and Palmer 1934), extraction of HA has been widely carried out using other animal tissues including human umbilical cord, rooster comb, and bovine synovial fluid. HA derived from animal tissues have naturally high molecular weights and the highest concentration ever reported was from rooster combs which can reach up to 7500 μg hyaluronan/g of that animal tissue (Fraser et al. 1997).

Today, although animal-derived HA still remains as an important resource for most HA-based products, alternative production processes have been sought for a number of reasons. First, the extraction processes have always experience technical limitations due to harsh extraction conditions that comes with grinding, acid treatment, and repeated extraction with organic solvents. This uncontrolled degradation technique greatly affects not only the yield but also the polydispersity (range of sizes) of HA (Boeriu et al. 2013). A second problem is that animal HA may still be bound to cellular proteins including hyaluronidase, a HA-specific binding protein (Fraser et al. 1997). These contaminant proteins are undesirable as there may be a chance that these will illicit an immune response. Moreover, there is a potential risk of contamination with nucleic acids, prions (bovine) and viruses (avian) which could result in the transmission of infectious disease (Shiedlin et al. 2004). Finally, extracting HA from animal tissues is costly as it takes considerable time to complete, is labour intensive and requires large facilities that can accommodate processes involved from collection of tissue from the animal to extraction and purification of HA. As a consequence of these technical and safety issues, biotechnological production of HA is seen as a preferred method of producing HA.

### Bacterial production of HA

Production of HA by bacterial fermentation has evolved steadily over the past two decades. In its early stage of development, Group A and C *Streptococci* that naturally produced HA were grown in fermenters and HA was purified. However, as these bacteria produce a number of toxins, alternative bacteria were sought. Once the genes that encode for the HA biosynthetic pathway were determined, a number of bacteria (*Bacillus, Agrobacterium, E. coli and Lactococcus*) were genetically modified to express these genes and produce HA. Subsequent work has focused on optimization of culture media and cultivation conditions (DeAngelis et al. 1993, 1998; Mao and Chen 2007; Wessels et al. 1991; Widner et al. 2005).

Currently, the *Bacillus* production system (*B. subtilis)* is a well-characterized Gram-positive microorganisms (along with Group A and C *Streptococci*) established as industrial workhorses for the production of various products, including HA (Novozymes). The expression constructs utilizing *hasA* gene from *S*. *equisimilis* in combination with overexpression of one or more of the three native *B*. *subtilis* precursor genes (homologous to *hasB, hasC and hasD* in *Streptococci*) were used. *B. subtilis* has highly developed biosynthetic capacity and capability to grow in industrial fermenters. Its system is free of exotoxins and endotoxins and, consequently, many products produced in this organism have been awarded a GRAS (*g*enerally recognized as safe) designation (Widner et al. 2005).

**Fig. 5:**
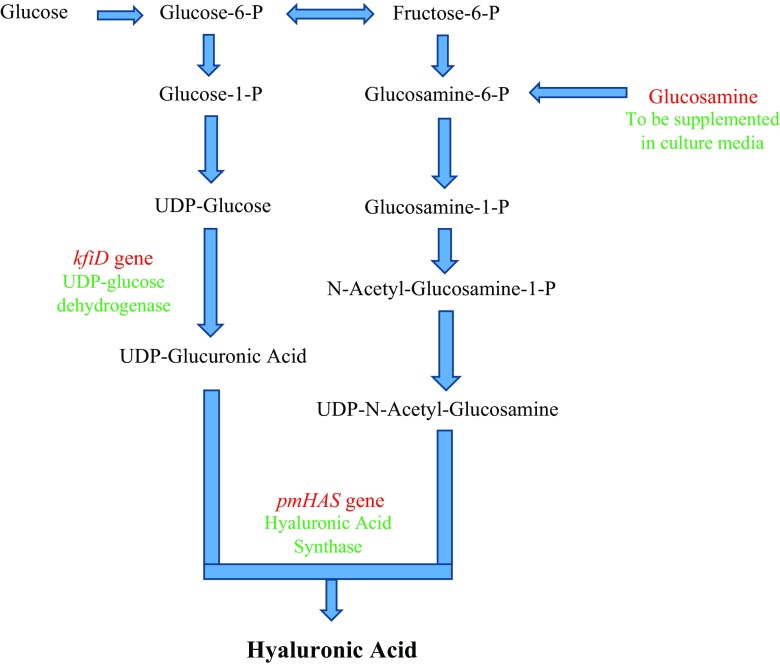
Overview of biosynthesis pathway for recombinant HA production in *E. coli*. In order to complete the HA biosynthetic pathway in the *E. coli*, expression of *kfiD* and *pmHAS* genes along with supplementation of glucosamine (with glucose) into the culture media are needed to allow the bacteria to produce HA (Mao et al. 2009)

While production of HA is commonly observed in Gram-positive bacteria, many Gram-negative bacteria, such as *E. coli* do not produce HA, as they either lack key enzymes of the biosynthetic pathway, or express components of the pathway at very low levels. Most strains of *E. coli* commonly used in laboratories, such as JM109, do not synthesise HA for these reasons. If, however, UDP-glucose dehydrogenase (*kfiD*) from *E. coli* K5 and HAS (*pmHAS*) from *P. multocida*, are expressed, it is possible to synthesise HA in *E. coli* strains such as JM109 (Mao et al. 2009). HA production was further enhanced in this system when combined with supplementation of the media with glucose and glucosamine (precursors of HA) (Fig. 5), during the induction process, with yields as high as 3.8 g/L and maximal molecular weight of 1.5 MDa.

### Cell-free production of HA (in vitro system)

While bacterial expression systems can produce HA in small scale fermenters, in larger systems the production of HA increases the viscosity of the media resulting in poor mixing and low oxygen mass transfer rate, thus often yields greater than 6–7 g/L are difficult to achieve (Liu et al. 2011). High polydispersity of HA produced in bacterial fermentation also remains a challenge as production of narrow polydispersity (or ideally, monodisperse) appears to be highly dependent on the culture conditions. Finally, as is the case for animal sources of HA, the bacterial cell itself can act as a source of contamination (nucleic acids, HA binding proteins, toxins, etc.) that may elicit an immune response (Boeriu et al. 2013). Due to the limitations associated with current mass production either from animal or bacterial sources, cell-free production system (in vitro) is seen as a desirable alternative.

Class I HAS family members are integral membrane proteins and thus they are lipid-dependent. Isolation and purification of this enzyme can be difficult as it requires active detergent to solubilise the Class I HAS-membrane-bound structure (De Luca et al. 1995; Tlapak-Simmons et al. 2005). Without the close association with phospholipid layer the function of Class I HAS enzyme would be impaired. Thus while HA production is possible, it cannot be achieved at a commercial scale.

The Class II HAS from *P. multocida* is amenable for cell free HA production as the enzyme is a peripheral (not an integral membrane protein) and does not need to be bound to the membrane in order to function. Furthermore, deletion studies have shown that removal of the membrane domains (residues 704–972) generates a soluble enzyme (known as pmHAS1-703) that retains the ability to synthesise HA (Jing and DeAngelis 2000). Using this cell-free system it is possible to produce HA with high molecular weight (~1–2 MDa) with low polydispersity (Jing and DeAngelis 2004). The processivity of this HAS could be controlled through the addition of HA oligomers (or acceptors) to the reaction mixture, which circumvented the rate-limiting step (formation of first glycosidic linkage) allowing subsequent sugars to be added rapidly to the ends of the oligomers, while the length of the HA polymer was shown to be inversely proportional to the abundance of oligomers. While high molecular weight HA can be achieved using this system, the total yield of HA remains very low.

## Future perspective

Given the widespread cosmetic and medical applications of HA, commercial interest in the rapid and safe production of HA remains strong. Cell-free and/or non-pathogenic bacterial expression systems are considered to be safer alternatives to existing production systems, though as discussed previously, are limited in their capacity to produce large amounts of HA. While genes encoding for Class I or Class II HAS enzymes cloned from diverse bacteria have successfully synthesised HA in novel non-pathogenic host bacteria such as *E. coli*, these enzymes have only ever been expressed independently (Mao et al. 2009; Yu and Stephanopoulos 2008). As both Class I and II HAS enzymes extend the growing HA polymer in unique ways, in theory these could act synergistically to produce HA. Such experiments should be possible in *E. coli* or other tractable bacterial expression systems.
